# Skin-resident T cells play an important role in controlling skin colonization of *Candidozyma (Candida) auris*

**DOI:** 10.1016/j.isci.2026.115862

**Published:** 2026-04-24

**Authors:** Jiajia Xie, Liping Yan, David Kadosh, Na Xiong

**Affiliations:** 1 Department of Microbiology, Immunology and Molecular Genetics, University of Texas Health Science Center San Antonio, 7703 Floyd Curl Drive, San Antonio, TX 78229, USA; 2 Department of Dermatology, The First Affiliated Hospital, Division of Life Sciences and Medicine, University of Science and Technology of China, Hefei, China; 3 Department of Medicine-Division of Dermatology and Cutaneous Surgery, University of Texas Health Science Center San Antonio, 7703 Floyd Curl Drive, San Antonio, TX 78229, USA

**Keywords:** immunology, cell biology

## Abstract

*Candidozyma auris* (formerly *Candida auris*) is an emerging multidrug-resistant fungal pathogen that can colonize the skin for a long time, enabling its prolonged transmission. Understanding the immune mechanisms that control skin colonization of *C. auris* is critical for the development of immune-based preventive and therapeutic strategies. In this study, we dissected the roles of T cells in controlling *C. auris* skin colonization using mouse models. We found that the inhibition of T cell infiltration into the skin had little effect on *C. auris* colonization. On the other hand, CCR10-knockout mice defective in the homeostatic establishment of skin-resident T cells had increased *C. auris* skin colonization despite enhanced IL-17^+^ T cell responses. Furthermore, we identified CD8^+^ skin-resident T cells as an important T cell population in controlling *C. auris* colonization. Together, our findings reveal that skin-resident but not infiltrating T cells play a dominant role in controlling *C. auris* skin colonization.

## Highlights

• Skin-resident T cells play a vital role in controlling *C. auris* skin colonization
• Both IL-17^+^ and IL-17^-^ skin-resident T cells help controlling *C. auris*
• Balanced T cell responses control *C. auris* and maintain skin tissue homeostasis
• Skin-resident CD8^+^ T cells are an important population in controlling *C. auris*

## Graphical abstract

**Figure:**
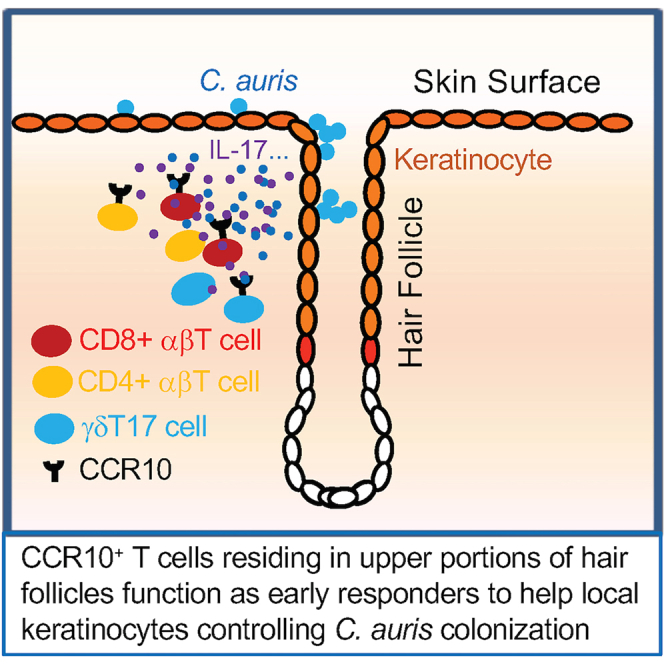

## Introduction

*Candidozyma auris* (formerly *Candida auris*) is an emerging multidrug-resistant (MDR) human fungal pathogen that poses an increased threat to public health worldwide.^1^ It was first isolated from a patient in Japan in 2009.^2^ Since then, *C. auris* has been found in dozens of countries across six continents.^3^ *C. auris* possesses a strong ability to colonize the skin surface and hair follicles for a long period of time, enabling it to serve as a reservoir for sustained person-to-person transmission in health care facilities and beyond.^3^^,^^4^^,^^5^^,^^6^ Prolonged colonization of *C. auris* in the skin could also increase the incidence of invasive infections that cause diseases in various tissues and organs, such as myocarditis, pericarditis, meningitis, osteomyelitis, and lead to high rates of mortality (∼40%) in patients with chronically ill and immunocompromised conditions.^3^^,^^7^^,^^8^^,^^9^ Since a high percentage of *C. auris* isolates are resistant to multiple currently used antifungal agents, infections are difficult to treat once there are outbreaks.^3^

Immune responses can control and eventually clear *C. auris* in immunocompetent hosts.^3^^,^^10^ Therefore, understanding immune mechanisms that control *C. auris* infections, particularly in the skin, is crucial to investigating pathogenesis and developing immune-based preventive and therapeutic strategies. Studies in recent years have started to uncover immune defense mechanisms against *C. auris* infections. Pathogen-associated molecular patterns (PAMPs) such as carbohydrates unique to cell walls of *C. auris* can be recognized by receptors such as macrophage mannose receptor and complement receptor-3 expressed by innate immune cells such as macrophages, dendritic cells (DCs), and neutrophils. However, innate immune responses to *C. auris* are blunted compared to those against *Candida albicans*, as demonstrated by the reduced migration and recruitment of phagocytes to *C. auris* infection sites and the reduced production of proinflammatory cytokines and killing by activated immune cells.^11^^,^^12^^,^^13^ This is at least partly because the unique mannan structure in cell walls of *C. auris* helps to evade immune detection and suppresses activation signals in innate immune cells.^14^^,^^15^

Investigation into immune mechanisms controlling *C. auris* skin colonization was only reported recently. Using a mouse model of topical *C. auris* application, Huang et al. first demonstrated that the coordinated activation of IL-17-producing γδT, αβT cells and innate lymphoid cells (ILCs) in the skin controls *C. auris* colonization, while mice deficient in T cells and ILCs have an impaired ability to control and clear *C. auris* colonization.^6^ IL-17-producing cells could exert their effects in controlling *C. auris* colonization through IL-17-induced signals on skin keratinocytes to promote the production of antimicrobial peptides (AMPs).^6^^,^^16^ IL-17 could also recruit additional immune cells, such as neutrophils, to the infection site. *C. auris* induces weaker IL-17^+^ T cell responses than *C. albicans* does in an intradermal infection model of mice,^17^ which might partially explain its prolonged skin colonization. A single-cell RNA-seq analysis identified multiple immune cell subsets, including phagocytic cells, DCs, T cells, and natural killer (NK) cells, accumulating at the intradermal injection site of *C. auris* infection.^15^ However, it is not clear how activation and localization of different T cell populations are regulated in the skin to control *C. auris* colonization. A previous study found that deficiency of Langerhans cells did not impair the immune regulation of topical *C. auris* colonization, suggesting that local T cell responses to *C. auris* colonization are independent of antigen-presenting functions of these skin-resident DCs.^6^

In this study, we dissected the roles of skin-resident and infiltrating T cells in controlling *C. auris* colonization in the skin using mouse models. Our study reveals that innate responses of skin-resident T cells, but not adaptive responses of infiltrating T cells, play a major role in controlling *C. auris* colonization. We further found that CCR10-dependent skin-resident T cells play an important role in controlling *C. auris* colonization in an IL-17-independent fashion. We identified skin-resident CD8^+^ T cells as an important T cell population in controlling *C. auris* colonization. Our findings provide insights into mechanisms underlying T cell regulation of *C. auris* colonization in the skin.

## Results

### Kinetic T cell responses are associated with controlling *C. auris* colonization in the skin

**Figure 1:**
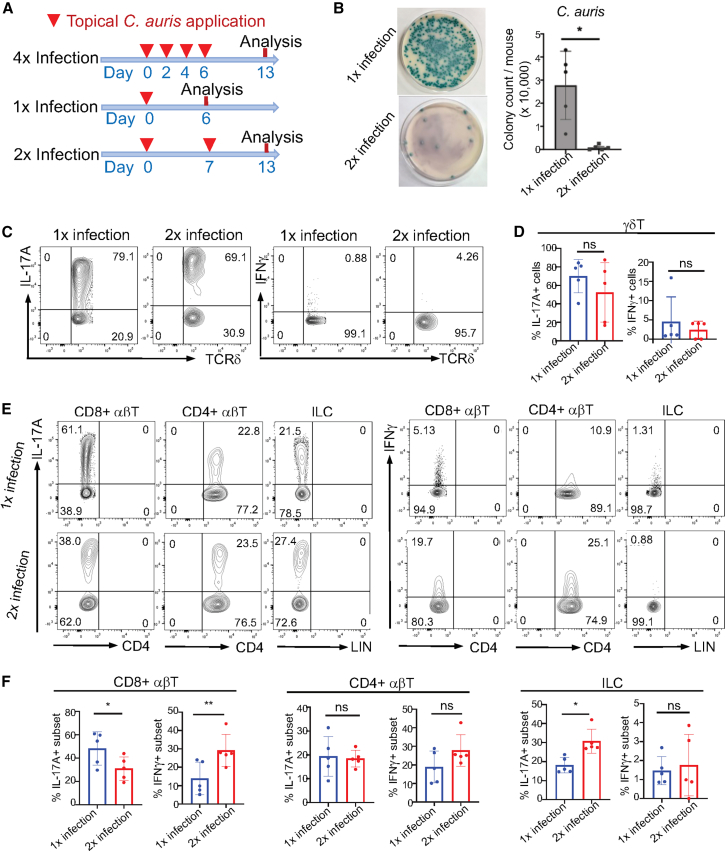
Kinetic activation of T cells is associated with the controlling skin colonization of *C. auris* (A) Schemes for various times of topical *C. auris* applications. (B) Representative images of colonies of *C. auris* grown from the skin digests of mice 6 days after 1× vs. 2× *C. auris* applications on CHROMagar *Candida* plates. Average numbers of colonies are shown in the graph on the left. One dot is one mouse. (C) Flow cytometric (FC) analysis of gated skin CD3^medium+^ γδT cells of *C. auris*-colonized mice for IL-17A or IFNγ 6 days after 1× vs. 2× *C. auris* applications. (D) Percentages of skin CD3^medium+^ γδT cells that express IL-17A or IFNγ 6 days after 1× vs. 2× *C. auris* applications. (E) FC analysis of skin CD8^+^ αβT, CD4^+^ αβT cells, and ILCs for IL-17A and IFNγ in mice 6 days after 1× vs. 2× *C. auris* applications. (F) Percentages of skin CD8^+^ αβT, CD4^+^ αβT cells, and ILCs that express IL-17A or IFNγ in mice 6 days after 1× vs. 2× *C. auris* applications. ns: not significantly different (*p* > 0.05), ∗*p* < 0.05 and ∗∗*p* < 0.01. Each dot represents one mouse. *N* = 5. Data were compiled from two separate experiments. The data are presented as mean ± SD. Statistical significance was determined by an unpaired *t* test.

To assess skin T cell responses to the colonization of *C. auris*, we first applied *C. auris* topically to the shaved back skin of wild-type (WT) mice every other day for four times (4×) (10^9^ *C. auris* cells/each application) and analyzed immune cells and fungal burdens in the skin on day 13 after the first application, in a similar manner as previously described^6^ (Figure 1A for the experimental scheme). There were differentially increased responses of IL-17A^+^ CD3^medium+^ γδT (γδT17) cells, CD4^+^ and CD8^+^ αβT (Th17 and Tc17) cells, and ILCs (ILC3s) in the skin of *C. auris*-colonized mice compared to those in uninfected naive mice (Figure S1A). Only a low number of *C. auris* were detected in the skin (Figure S1B), consistent with the notion that activated immune cells, including IL-17-producing cells, efficiently control *C. auris* skin colonization.^6^ However, the efficient control also makes it hard to dissect processes of T cell activation involved in response to *C. auris* colonization.

We then modified infection procedures to correlate T cell responses in the skin with controlling *C. auris* colonization at early and late time points of infection after one-time (1×) or two-time (2×) topical applications (Figure 1A). There was a 7-day interval in the 2× application scheme, and mice were analyzed 6 days after 1× or 2× applications (Figure 1A). The number of *C. auris* in the skin was drastically lower 6 days after 2× applications than after 1× application (Figure 1B), suggesting that immune cells activated by the first *C. auris* application provide efficient control against colonization of the second *C. auris* application. Comparison of T cells in the skin 6 days after 1× vs. 2× *C. auris* applications revealed that while percentages of IL-17A^+^ γδT cells remained similar at the early and late time points (Figures 1C and 1D), there were increased percentages of IFNγ^+^ αβT cells associated with decreased percentages of IL-17A^+^ αβT cells, particularly in CD8^+^ αβT cells, at the later time point (Figures 1E and 1F). On the other hand, there were increased percentages of IL-17A^+^ ILCs at the later time point (Figures 1E and 1F).

### Blockage of T cell infiltration into the skin by FTY720 does not significantly affect host regulation of *C. auris* colonization

**Figure 2:**
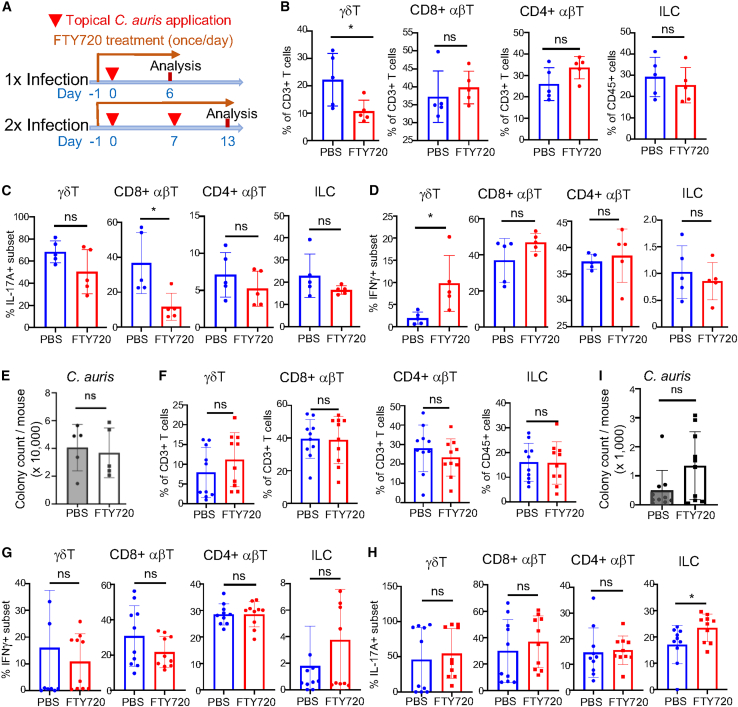
Blockage of T cell infiltration into the skin by FTY720 does not significantly affect the host regulation of *C. auris* colonization (A) Schemes of *C. auris* applications and FTY720 treatments. (B) Percentages of indicated subsets of T cells and ILCs in the skin of FTY720-treated and control PBS-treated mice 6 days after 1× *C. auris* application. (C and D) Percentages of skin γδT, CD8^+^ αβT, CD4^+^ αβT cells, and ILCs that express IL-17A (C) or IFNγ (D) in FTY720- and PBS-treated mice 6 days after 1× *C. auris* application. (E) Average numbers of colonies of *C. auris* in the skin of FTY720- vs. PBS-treated mice 6 days after 1× *C. auris* application. (F) Percentages of indicated subsets of T cells and ILCs in the skin of FTY720- vs. PBS-treated mice 6 days after 2× *C. auris* applications. (G and H) Percentages of skin γδT, CD8^+^ αβT, CD4^+^ αβT cells, and ILCs that express IFNγ (G) or IL17A (H) in FTY720- and PBS-treated mice 6 days after 2× *C. auris* applications. (I) Average numbers of colonies of *C. auris* in the skin of FTY720- vs. PBS-treated mice 6 days after 2× *C. auris* applications. One dot is of one mouse. B-E, *N* = 5; data were compiled from two separate experiments. (F–H), *N* = 10; data were compiled from three separate experiments. The data are presented as mean ± SD. ns: not significantly different (*p* > 0.05), ∗*p* < 0.05. Statistical significance was determined by an unpaired *t* test.

We then treated mice with FTY720 to first distinguish relative contributions of responses of skin-resident vs. infiltrating T cells and ILCs in the 1× and 2× application schemes (Figure 2A). FTY720 blocks the migration of T cells and ILCs from circulation into the skin by impairing sphingosine-1-phosphate receptor 1 (S1PR1)-mediated signals.^18^^,^^19^ Therefore, only responses of skin-resident T cells and ILCs would be involved in controlling *C. auris* skin colonization in FTY720-treated mice.

On day 6 after 1× *C. auris* application, FTY720-treated mice had significantly reduced percentages of CD3^medium+^ γδT cells, which included most γδT17 cells, compared to control PBS-treated mice (Figures 2B and 2C). This result suggests that infiltrating γδT17 cells accounted for about half of the total γδT17 cells in the skin colonized by *C. auris*. In addition, FTY720-treated mice had decreased percentages of IL-17A+ skin αβT cells, particularly in the CD8^+^ subset, compared to PBS-treated mice (Figure 2C), suggesting that a significant fraction of Tc17 cells also migrated into the skin in response to *C. auris* colonization. There was a relatively increased percentage of IFNγ^+^ γδT cells in FTY720-treated mice, although they represented a minor fraction of total γδT cells (Figure 2D). There was no statistical difference in total, IL-17A^+^ or IFNγ^+^ ILCs in FTY720-treated and control mice (Figures 2B–2D). These results indicate that a significant percentage of IL-17A^+^ γδT and αβT cells migrate into the skin in response to the *C. auris* colonization, which is blocked by the FTY720 treatment. Surprisingly, despite the blockage of migration of these IL-17-producing T cells into the skin, there was no statistical difference in levels of *C. auris* in the skin of FTY720- vs. PBS-treated mice (Figure 2E), revealing that responses of skin-resident, but not infiltrating, T cells play a critical role in controlling early colonization of *C. auris*.

We also assessed the effects of the FTY720 treatment on T cell responses and *C. auris* burdens in the skin 6 days after 2× *C. auris* applications (13 days after the first application) (Figure 2A). There were no significant differences in percentages of total, IL-17A^+^ or IFNγ^+^ γδT, CD8^+^ αβT, CD4^+^ αβT cells in FTY720- vs. PBS-treated mice 6 days after 2× applications (Figures 2F–2H), suggesting that responses of skin-resident T cells might compensate for blockage of infiltrating T cells in FTY720-treated mice at the late time point. There were also no significant differences in total and IFNγ^+^ ILCs, while IL-17A^+^ ILCs were increased in FTY720-treated mice compared to PBS-treated controls (Figures 2F–2H). In addition, although there might be a trend of increase in levels of *C. auris* in the skin of FTY720-treated mice compared to PBS-treated mice on day 6 after 2× applications, they were both greatly reduced (>20-fold) from levels of *C. auris* on day 6 after 1× application (Figures 2I vs. 2E). These results further confirmed that responses of skin-resident T cells play a dominant role in controlling *C. auris* colonization in the skin.

### CCR10-dependent skin-resident T cells play an important role in controlling skin colonization of *C. auris* in an IL-17-independent fashion

To further dissect the roles of skin-resident T cells in controlling *C. auris* colonization, we infected a strain of CCR10-KO/EGFP-knockin (KI) mice with the topical *C. auris* application. CCR10 is a chemokine receptor expressed by most skin-resident T cells and is critical for their homeostatic establishment through interaction with its ligand CCL27, expressed by keratinocytes.^20^^,^^21^ Homozygous CCR10-KO/EGFP-KI (CCR10^EGFP/EGFP^, or CCR10^−/−^ for simplicity) mice have dysregulated skin-resident T cells compared to heterozygous CCR10-KO/EGFP-KI (CCR10^+/EGFP^ or CCR10^+/−^) mice, characterized mostly by the reduction of CD8^+^ and CD4^+^ αβT cells but not IL-17A^+^ αβT or γδT cells.^20^^,^^22^^,^^23^

**Figure 3:**
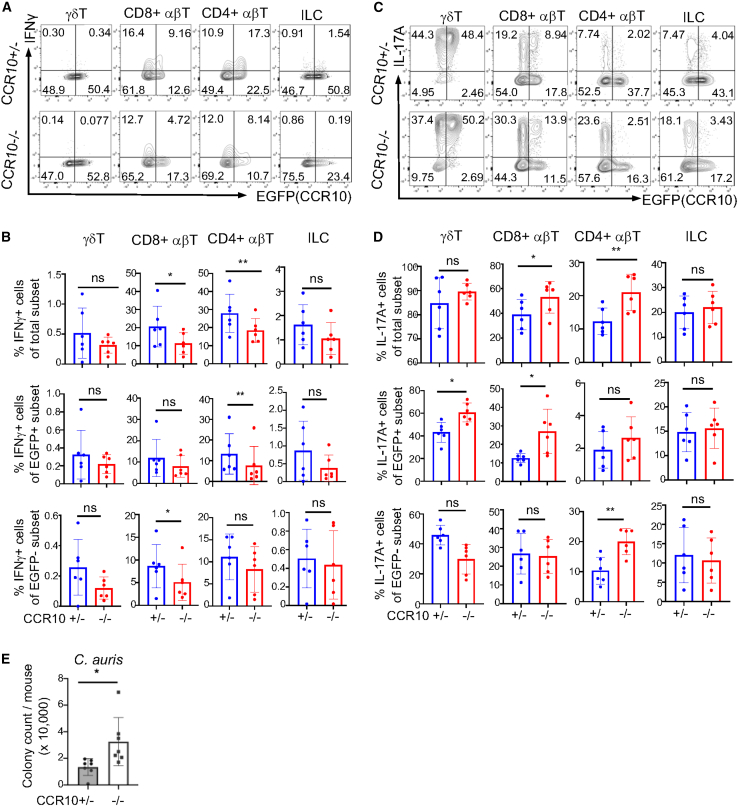
CCR10-dependent skin-resident T cells play an important role in controlling the skin colonization of *C. auris* CCR10^+/EGFP^ and CCR10^EGFP/EGFP^ mice were infected with 1× topical *C. auris* application and analyzed on day 6 post infection. In CCR10^+/EGFP^ mice, the coding sequence of one CCR10 allele is replaced by an EGFP-coding sequence for the purpose of reporting CCR10 expression with EGFP, while in CCR10^EGFP/EGFP^ mice, EGFP^+^ cells are “CCR10^+^ wannabe” cells that are supposed to express CCR10 but cannot because coding sequences of both CCR10 alleles are replaced by EGFP.^22^ (A and C) FC analysis of skin γδT, CD8^+^ αβT, CD4^+^ αβT cells, and ILCs of CCR10^+/EGFP^ and CCR10^EGFP/EGFP^ mice for the expression of IFNγ and EGFP (CCR10) (A) or IL-17A and EGFP(CCR10) (C). (B and D) Percentages of total (top row), EGFP(CCR10)^+^ (middle), and EGFP(CCR10)^-^ (bottom) skin γδT, CD8^+^ αβT, CD4^+^ αβT cells, and ILCs that express IFNγ (B) or IL-17A (D) in CCR10^+/EGFP^ vs. CCR10^EGFP/EGFP^ mice. (E) Average numbers of colonies of *C. auris* in the skin of CCR10^+/EGFP^ and CCR10^EGFP/EGFP^ mice. One dot is of one mouse. (B–D), *N* = 6; data were compiled from two separate experiments. (E) *N* = 7; data were compiled from three separate experiments. The data are presented as mean ± SD. ns: not significantly different (*p* > 0.05), ∗*p* < 0.05, and ∗∗*p* < 0.01. Statistical significance was determined by an unpaired *t* test.

On day 6 after 1× topical *C. auris* application, about half of IFNγ^+^ skin CD8^+^ and CD4^+^ αβT cells of CCR10^+/EGFP^ mice expressed CCR10(EGFP) while they had few IFNγ^+^ γδT cells or ILCs (Figure 3A). Compared to CCR10^+/EGFP^ mice, CCR10^EGFP/EGFP^ littermates had significantly reduced percentages of IFNγ^+^ CD8^+^ and CD4^+^ skin T cells (Figures 3A and 3B). On the other hand, most skin IL-17A^+^ CD8^+^, CD4^+^ T cells and ILCs did not express CCR10(EGFP), while half of skin γδT17 cells were CCR10(EGFP)^+^ in CCR10^+/EGFP^ mice (Figure 3C). Compared to CCR10^+/EGFP^ mice, CCR10^EGFP/EGFP^ littermates had significantly increased percentages of IL-17A^+^ CD8^+^ and CD4^+^ skin T cells, while they had similar percentages of IL-17A^+^ γδT cells and ILCs (Figures 3C and 3D). Associated with the increased IL-17^+^ T cell responses, CCR10^EGFP/EGFP^ mice had more severe skin inflammation than CCR10^+/EGFP^ mice (Figures S2A–S2C). However, despite their enhanced IL-17A^+^ T cell responses, CCR10^EGFP/EGFP^ mice had significantly higher levels of *C. auris* in the skin than their CCR10^+/EGFP^ littermate controls (Figure 3E). These results suggest that CCR10-dependent skin-resident T cells play an important role in restricting the early colonization of *C. auris* in the skin, independent of IL-17 responses.

We also compared CCR10^+/EGFP^ and CCR10^EGFP/EGFP^ mice on day 13 after the first application in the repeated *C. auris* topical application scheme. Similar to results on day 6 after 1× application, CCR10^EGFP/EGFP^ mice also had increased IL-17A^+^ T cell responses compared to CCR10^+/EGFP^ littermate mice on day 13 (Figure S2D). The increased IL-17A^+^ T cell responses in CCR10^EGFP/EGFP^ mice on day 13 became even more prevalent because there were significantly higher percentages of IL-17A^+^ cells in every T cell subset (γδT, CD8^+^ αβT, and CD4^+^ αβT) in CCR10^EGFP/EGFP^ mice than in CCR10^+/EGFP^ mice (Figure S2D). CCR10^EGFP/EGFP^ and CCR10^+/EGFP^ mice had similarly low levels of *C. auris* in the skin on day 13 (Figure S2E), both being greatly reduced from levels on day 6 (Figure 3E). These results suggest that the enhanced IL-17A^+^ T cell responses in CCR10^EGFP/EGFP^ mice catch up at the later time point in controlling *C. auris* colonization.

### Increased IL-17A^+^ T cell responses in CCR10-knockout mice help to compensate for impaired control of *C. auris* colonization by skin-resident T cells

**Figure 4:**
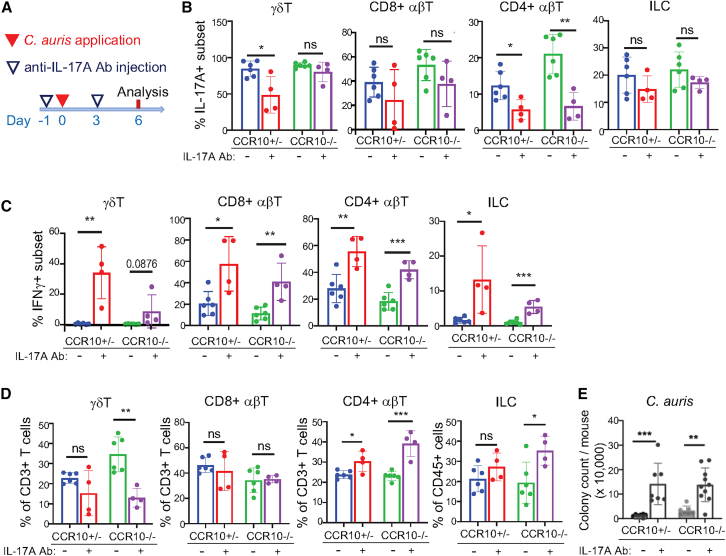
Increased IL-17A^+^ T cell responses help to compensate for the impaired regulation of *C. auris* colonization by skin-resident T cells in CCR10-KO mice (A) Schemes of *C. auris* application and anti-IL-17A antibody (Ab) treatments. (B and C) Percentages of skin γδT, CD8^+^ αβT, CD4^+^ αβT cells, and ILCs that express IL-17A (B) or IFNγ (C) in anti-IL-17A antibody-treated and untreated CCR10^+/EGFP^ and CCR10^EGFP/EGFP^ mice 6 days after 1× *C. auris* application. (D) Percentages of skin γδT, CD8^+^ αβT, CD4^+^ αβT, and ILCs in anti-IL-17A antibody-treated and untreated CCR10^+/EGFP^ and CCR10^EGFP/EGFP^ mice 6 days after 1× *C. auris* application. (E) Average numbers of colonies of *C. auris* in the skin of anti-IL-17A antibody-treated and untreated CCR10^+/EGFP^ and CCR10^EGFP/EGFP^ mice 6 days after 1× *C. auris* application. (B–D), *N* = 6 for the CCR10^+/EGFP^ group and 4 for the CCR10^EGFP/EGFP^ group. E, *N* = 7 in each group. Data were compiled from two separate experiments. The data are presented as mean ± SD. ns: not significantly different (*p* > 0.05), ∗*p* < 0.05, ∗∗*p* < 0.01, and ∗∗∗*p* < 0.001. Statistical significance was determined by an unpaired *t* test.

To address further whether the increased IL-17A^+^ T cell responses in CCR10^EGFP/EGFP^ mice helped to compensate for the defective regulation of *C. auris* colonization by CCR10-dependent skin-resident T cells, we treated CCR10^EGFP/EGFP^ and CCR10^+/EGFP^ mice with neutralizing anti-IL-17A antibodies during the course of a 1× *C. auris* skin colonization scheme (Figure 4A). CCR10^+/EGFP^ and CCR10^EGFP/EGFP^ mice treated with anti-IL-17A antibodies both had reduced percentages of IL-17A^+^ γδT, CD8^+^ αβT, CD4^+^ αβT cells to various degrees compared to untreated mice (Figure 4B). On the other hand, anti-IL-17A antibody-treated CCR10^+/EGFP^ and CCR10^EGFP/EGFP^ mice had increased percentages of IFNγ^+^ subsets in γδT, CD8^+^ αβT, CD4^+^ αβT cells, and ILCs compared to untreated mice (Figure 4C). As a result, the percentage of CD3^medium+^ γδT cells, which include most γδT17 cells, was significantly reduced while percentages of CD4^+^ αβT cells increased in anti-IL-17A antibody-treated mice (Figure 4D). Associated with the neutralization of IL-17A and reduction of IL-17A^+^ T cells, there were highly increased levels of *C. auris* in the skin of anti-IL-17A antibody-treated CCR10^+/EGFP^ and CCR10^EGFP/EGFP^ mice (Figure 4E). These results support the notion that increased IL-17A-producing T cells in CCR10^EGFP/EGFP^ mice help to control *C. auris* colonization but they are not sufficient to completely compensate for defective responses of CCR10-dependent skin-resident T cells.

### Skin-resident CD8^+^ T cells play an important role in controlling *C. auris* colonization

**Figure 5:**
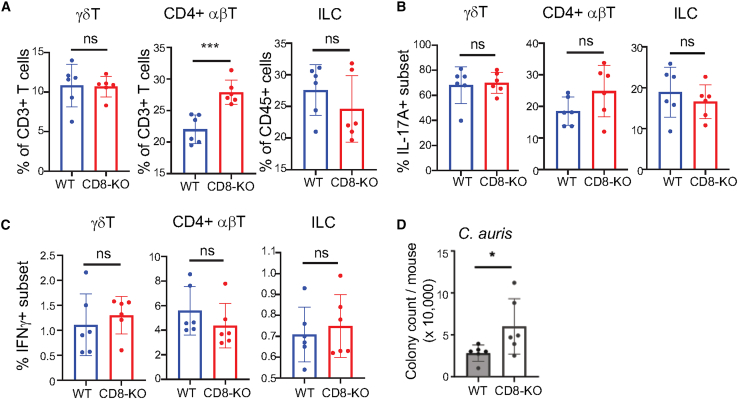
Skin-resident CD8^+^ T cells play an important role in controlling *C. auris* colonization in the skin (A) Percentages of skin γδT, CD4^+^ αβT cells, and ILCs in CD8-KO and WT mice 6 days after 1× *C. auris* application. (B and C) Percentages of skin γδT, CD4^+^ αβT cells, and ILCs that express IL-17A (B) or IFNγ (C) in CD8-KO and WT mice 6 days after 1× *C. auris* application. (D) Average numbers of colonies of *C. auris* in the skin of CD8-KO and WT mice 6 after 1× *C. auris* application. One dot is of one mouse. *N* = 6. Data were compiled from two separate experiments. The data are presented as mean ± SD. ns: not significantly different (*p* > 0.05), ∗*p* < 0.05 from ∗∗*p* < 0.01. Statistical significance was determined by an unpaired *t* test.

Since CD8^+^ skin-resident T cells are a major subset of T cells that are impaired in the skin of CCR10^EGFP/EGFP^ mice,^20^ we tested whether they played an important role in controlling *C. auris* skin colonization using CD8a-knockout (CD8-KO) mice, which are deficient of CD8^+^ T cells.^24^ To prevent inference of infiltrating T cells, we treated CD8-KO and control WT mice with FTY720 during the course of 1× *C. auris* topical colonization. Compared to WT mice, CD8-KO mice had compensatorily increased percentages of CD4^+^ αβT cells, while they had similar γδT cells and ILCs in the skin on day 6 after 1× *C. auris* application (Figure 5A). Percentages of IL-17A^+^ CD4^+^ αβT cells, γδT cells, and ILCs in the skin of CD8-KO mice were similar to those of WT controls (Figure 5B). Percentages of IFNγ^+^ CD4^+^ αβT cells, γδT cells, and ILCs were also similar in CD8-KO and WT mice (Figure 5C). However, even with the compensatorily increased CD4^+^ T cells, CD8-KO mice had significantly higher levels of *C. auris* in the skin than WT mice (Figure 5D). These results demonstrated that skin-resident CD8^+^ T cells play an important role in controlling *C. auris* colonization.

## Discussion

Due to its rapid emergence, high mortality and multidrug resistance, *C. auris* was placed among the most severe Critical Priority Group of the World Health Organization (WHO) fungal priority pathogens list.^25^ Considering that persistence of *C. auris* in skin is a major cause for its sustained transmission and increased incidences of severe systemic infections, understanding how immune cells control *C. auris* skin colonization will help the development of immune-based strategies to prevent and treat the infection. In this report, we found that skin-resident, but not infiltrating, T cells play a major role in controlling *C. auris* skin colonization. We also found that both IL-17-dependent and independent responses of skin-resident T cells are required to control *C. auris* colonization and maintain skin tissue homeostasis, while imbalanced T cell responses led to impaired control of C. auris and increased skin inflammation in CCR10-KO mice. Our findings suggest skin-resident T cells as an important immune cell population for targeting in the development of immune-based strategies to prevent and eradicate *C. auris* from its colonization sites in the skin. However, critical questions on molecular mechanisms mediating functions of skin-resident T cells in controlling *C. auris* colonization, as well as regulating their activation, need to be addressed before the findings can be translated into clinical usage.

In terms of molecular mechanisms mediating functions of skin-resident T cells in controlling *C. auris* colonization, both our current study and a previous report suggest that IL-17A plays a role.^6^ IL-17A can stimulate the production of AMPs of skin keratinocytes to inhibit *C. auris* colonization.^6^ Our study suggests that IL-17-independent mechanisms are also required for skin-resident T cells to control *C. auris* colonization, although effector molecules involved in the IL-17A-independent mechanisms are yet to be identified. In the future, we could perform RNA-seq analyses of skin-resident T cells of CCR10^+/EGFP^ and CCR10^EGFP/EGFP^ mice to identify potentially important effector molecules to further dissect their functional mechanisms in controlling *C. auris* colonization. Considering most skin-resident T cells express CCR10 and preferentially reside in regions close to the upper portions of hair follicles,^20^^,^^21^ effector molecules expressed by skin-resident T cells likely act on follicular keratinocytes nearby to enhance their abilities against *C. auris* colonization or directly inhibit *C. auris* in hair follicles.

Molecular mechanisms regulating the activation of skin-resident T cells in response to *C. auris* colonization also require further study. All skin-resident T cells are pre-activated T cells with effector/memory-like phenotypes, which could be activated in an innate fashion by alterations of molecular cues of the local tissue they reside in.^26^^,^^27^^,^^28^^,^^29^^,^^30^^,^^31^^,^^32^ Our finding that blockage of infiltrating T cells by FTY720 does not significantly affect *C. auris* colonization suggests that innate-like responses of skin-resident T cells, but not adaptive responses of infiltrating T cells, play a major role in controlling *C. auris* colonization. Likely, *C. auris*-colonized keratinocytes generate molecular cues that directly activate nearby skin-resident T cells, which in turn produce effector molecules to help keratinocytes against *C. auris*. Consistent with this notion, it was previously reported that IL-23 produced by keratinocytes can activate skin-resident IL-17^+^ γδT cells to control bacterial and fungal pathogen infections.^32^^,^^33^ Similar mechanisms are likely involved in the activation of skin-resident IL-17^+^ γδT cells in response to *C. auris* colonization. However, molecular mechanisms required for the activation of skin-resident αβT cells might be different. In addition, how keratinocytes sense *C. auris* colonization and activate skin-resident T cells is not clear. The C-type lectin receptor adaptor molecule Card9 was found to promote IL-17^+^ T cell responses to some fungal pathogens but not to *C. auris*,^6^^,^^34^ suggesting different mechanisms for keratinocytes to sense *C. auris* colonization.

Considering commensal bacteria play an important role in shaping skin-resident T cell repertoire, they likely also have a role in the regulation of *C. auris* colonization. In fact, in our experiments of the 4× repeated topical *C. auris* colonization scheme, we consistently obtained greatly reduced levels of the same strain of *C. auris* in the skin of WT mice compared to those previously reported.^6^ While reasons for the different *C. auris* levels in different animal facilities could be multiplex; commensal bacteria are an important factor to consider. Potentially, commensal bacteria could activate skin-resident IL-17A^+^ T cells and/or other immune cells to suppress *C. auris* colonization. Alternatively, commensal bacteria could compete directly with *C. auris* for niches in its colonization sites in hair follicles.^35^^,^^36^^,^^37^ Commensal bacteria could potentially produce AMPs to inhibit the growth of *C. auris*.^38^ How commensal bacteria coordinate with skin-resident T cells and other immune cells in response to and control *C. auris* colonization should be exploited further for the development of translational applications in the prevention and treatment of the fungal infection.

Various models of *C. auris* infection were used to address specific questions from skin colonization to invasive infections and pathology.^17^^,^^39^ Topical *C. auris* application is considered more closely to mimic the natural process of skin colonization.^6^ There are different skin T cell responses after one vs. repeated *C. auris* topical applications, suggesting that kinetic T cell responses are associated with *C. auris* colonization. We found that while IL-17A^+^ T cells are dominant at early time points after 1× *C. auris* colonization, there are increased percentages of IFNγ^+^ T cells at a later time point after repeated *C. auris* colonization. A recent report found that IFNγ enhances re-infection of *C. auris* in an intradermal injection model of infection.^40^ In future studies, we could use topical application schemes to further dissect the roles of skin-resident T cells and their effector molecules in primary *C. auris* infection and re-infection. Particularly, while our current study suggests that innate responses of skin-resident T cells play a dominant role in controlling early *C. auris* colonization, it is possible that antigen-specific T responses might play a more important role against re-infection after *C. auris*-specific skin-resident memory T cells are established in response to primary *C. auris* infection or immunization.

Our finding of increased skin inflammation in *C. auris*-colonized CCR10-KO mice provides evidence supporting the association of dysregulated immune responses to fungal pathogen infections and development of skin inflammatory diseases. It was suggested that while IL-17^+^ T cell responses are important in controlling commensal fungi or fungal pathogens, they can promote tissue inflammatory diseases in predisposed hosts.^41^^,^^42^^,^^43^ In CCR10-KO mice that are deficient of skin-resident T cells, there is compensatorily enhanced activation of IL-17A^+^ T cells to help control *C. auris* colonization but the over-active IL-17A^+^ T cell responses also cause more severe skin inflammatory symptoms. Consistent with this finding, we previously found that over-active IL-17A^+^ T cell responses also increase skin inflammation in CCR10^EGFP/EGFP^ mice in an imiquimod-induced model of psoriasis.^44^ These results indicate that imbalanced T cell responses in CCR10-KO mice lead to impaired control of pathogen infections and dysregulated skin immune homeostasis. Further investigation into mechanisms of enhanced activation of IL-17-producing T cells in *C. auris*-colonized CCR10-KO mice could help define the pathogenesis of dysregulated immune responses to fungal infections in the development of skin inflammatory diseases.

### Limitations of the study

The current study used a single *C. auris* strain (CDC #0383, African Clade) in all experiments. Given the known genetic and phenotypic diversity among different clades of *C. auris*, there is a limitation in extrapolating our findings to all other clades and strains. The generalizability of our findings could be expanded by using additional strains of *C. auris* of different clades. In addition, while our experiments using CCR10-KO mice provided strong evidence supporting the roles of CCR10-regulated skin-resident T cells in controlling *C. auris* colonization, it is possible that CCR10 could regulate other cells of the skin in addition to T cells. Cell-specific CCR10-KO mice could be used to further dissect the roles of CCR10 in regulated skin-resident T cells and other skin cells in response to *C. auris* colonization. Similarly, while our study of CD8-KO mice provided strong evidence for an important role of CD8^+^ skin-resident T cells in controlling *C. auris* colonization, using inducible CD8^+^ T cell knockout mice could avoid confounding effects resulting from the germline deletion of the CD8a gene and provide a more precise assessment of the functions of CD8^+^ T cells in controlling *C. auris* colonization. Our studies in this report focused on skin-resident cells. However, how many other immune cell populations, such as DCs, macrophages, mast cells, and neutrophils, are involved in response to *C. auris* colonization also needs more investigation. Our findings on the role of skin-resident T cells in controlling *C. auris* colonization would also need to be verified in human subsets for the development of immune-based preventive and therapeutic strategies against the fungal pathogen infection and transmission.

## Resource availability

### Lead contact

Further information and requests for resources and reagents should be directed to and will be fulfilled by the lead contact, Dr. Na Xiong (xiongn@uthscsa.edu).

### Materials availability

CCR10-knockout/EGFP-knockin mice were previously generated in the lab and are available upon request of reagents from the lead contact.

### Data and code availability

• All data reported in this paper will be shared by the lead contact upon request.
• This paper does not report original code.
• Any additional information required to reanalyze the data reported in this paper is available from the lead contact upon request.

## Acknowledgments

The research is supported by institutional funds (to JJM and NX). We thank the University of Texas Health Science Center at San Antonio histology and immunohistochemistry core facility, as well as the flow cytometry core facility, for excellent technical support. The flow cytometry core facility is supported by the Mays Cancer Center 10.13039/100000002NIH/10.13039/100000054NCI grant P30 CA054174 and the 10.13039/100000002NIH/10.13039/100006108National Center for Advancing Translational Sciences grant UL1 TR002645.

## Author contributions

J.X. performed experiments, analyzed data, and wrote the manuscript. L.Y. performed experiments, analyzed data, and wrote the manuscript. D.K. initiated the study and provided critical reagents. N.X. designed experiments, analyzed data, wrote the manuscript, and supervised the study.

## Declaration of interests

The authors declare no competing interests.

## STAR★Methods

### Key resources table

**Table:** 

REAGENT or RESOURCE	SOURCE	IDENTIFIER
**Antibodies**		

Anti-mouse CD45, APC-cy7	Biolgend	Cat # 103116: RRID:AB_312981
Anti-mouse TCRδ, Percpcy5.5	Biolgend	Cat # 118118; RRID:AB_10612756
Anti-mouse TCRδ, PE-cy7	Biolgend	Cat # 118124; RRID:AB_11204423
Anti-mouse CD3, BV5421	Biolgend	Cat # 155617; RRID:AB_2832541
Anti-mouse CD4, BV650	Biolgend	Cat # 100469; RRID:AB_2783035
Anti-mouse IL-17A, PE	Biolgend	Cat # 506904; RRID:AB_315464
Anti-mouse IFNγ, PE-cy7	BD Biosciences	Cat # 557649; RRID:AB_396766
Anti-mouse CD19, PE-cy7	Biolgend	Cat # 152417; RRID:AB_2927870
Anti-mouse IFNγ, AF647	BD Biosciences	Cat # 505814
Anti-mouse Live and Dead, BV510	Invitrogen	Cat # 2942302
Anti-mouse Streptavidin, PE-CF594	BD Biosciences	Cat # 562284: RRID:AB_11154598
Anti-mouse lineage (Lin)	Miltenyi Biotec	Cat # 5230206109
IL-17A neutralization antibody	Bio X Cell	Cat # BE0173; RRID:AB_10950102
IFNγ neutralization antibody	Bio X Cell	Cat # BE0055; RRID:AB_1107694
isotype control Rat IgG1 antibody	Bio X Cell	Cat # BE0088; RRID:AB_1107775
Anti-mouse CD3, APC	Biolgend	Cat # 100236; RRID:AB_2561456
Rabbit anti-*Candida albicans* antibody	Abcam	Cat # ab252746
Goat anti-Rabbit IgG, Alexa Fluor™ 750	Invitrogen	Cat # A-21039
Rabbit IgG isotype Control	Invitrogen	Cat # 02-6102
GMS staining kit	Newcomer Supply	Cat # 9121 A
CHROMagar *Candida* plates	Hardy Diagnostics	Cat #G343

**Experimental models: Organisms/strains**		

CD8-KO mice	The Jackson Lab	Strain # 002665
CCR10-KO/EGFP-KI mice	Generated in lab	None

### Experimental model and study participant details

#### Mice

CCR10-knockout(KO)/EGFP-knockin (KI) mice were previously described.^22^ CD8a-knockout mice were purchased from Jackson Lab (#002665).^24^ All mice were on the C57BL/6 genetic background and were raised in the SPF conditions. Sex- and age-matched male and female mice were used. Littermates were used when possible. All mouse experiments were performed in accordance with protocols approved by Institutional Animal Care and Use Committees of University of Texas Health Science Center at San Antonio (IACUC# 20190024AR).

### Method details

#### Topical application of *C. auris* in mice

*C. auris* (CDC #0383, African Clade)^6^ was grown in Yeast Extract-Peptone-Dextrose (YPD) medium at 30°C in a shaker for two days (200 rpm). Concentration of the *C. auris* culture was then determined based on optical density (OD) at 600 nm using a spectrophotometer. *C. auris* cells were harvested, washed and resuspended in PBS at the concentration of 5 × 10^9^ cells/mL for the topical application in mice. One or two days prior to topical application, dorsal hairs of mice were shaved and cleared by Nair. 1 × 10^9^ *C. auris* cells (in 200 μL PBS) were applied to the back skin of mice anesthetized with isoflurane, as reported previously.^6^ Times and frequencies of *C. auris* application are specifically indicated in relevant figures in the results section.

#### Digestion of skin tissue for single cell preparations

Experimental procedures were performed as we previously described.^20^ In brief, mouse skin was shaved manually and depilated with Nair. The adipose layer of the skin was removed. Skin was minced and digested for 2 h in a DMEM solution containing 4% BSA (Calbiochem), 8 mg/mL Collagenase I (Worthington), 4 mg/mL Collagenase IV (Worthington), and 2 mg/mL Hyaluronidase (Sigma). One and a half hours into the digestion, 0.0001% DNase (Sigma-Aldrich) was added to the solution. Single cell suspensions of the skin digests were used directly for quantification of *C. auris* in the skin and enriched further for immune cells.

#### Quantification of *C. auris*

One-tenth of the skin digests were collected, centrifuged and suspended in PBS. Total or 1/10 of the suspension was plated on CHROMagar *Candida* plates and incubated at 30°C for 3 days. Numbers of *C. auris* colonies were counted and their identities were verified using PCR. The total number of *C. auris* in the skin of each mouse was calculated based on the numbers of *C. auris* colonies and dilution factors.

#### PCR verification of *C. auris*

To verify the identity of *C. auris* cultured on CHROMagar *Candida* plates, colonies were picked and used for DNA preparation and PCR reactions. The PCR mixtures were prepared in a total volume of 25 μL, including 16.375 μL of ddH_2_O, 5 μL of 5xPCR Buffer, 0.5 μL of dNTP, 0.5 μL of each *C. auris*-specific primer, 0.125 μL of Taq polymerase, and 2 μL of *C. auris* DNA. The primer sequences are CauF 5′-CGCACATTGCGCCTTGGGGTA-3′and CauR 5′-GTAGTCCTACCTGATTTGAGGCGAC-3′, which are specific for a 5.8 S ribosomal RNA gene of *C. auris*.^45^ The PCR started with an initial denaturation for 3 min at 95°C, followed by 30 cycles of 20 s at 95°C, 20 s at 68°C, and 20 s at 72°C.

#### Enrichment of skin immune cells for flow cytometric analysis

Single cell suspensions of skin digests were enriched for immune cells by a 40% over 80% Percoll gradient as previously described.^20^ For immune staining of surface molecules, cells were incubated with properly fluorescently labeled antibodies in a PBS buffer containing 3% FBS for 30–45 min at 4°C. For intracellular cytokine staining, cells were stimulated with PMA, ionomycin and Brefeldin A for 4 h, followed by staining for surface molecules and then intracellular staining for cytokines. Stained cells were analyzed on BD LSRII or BD LSRFortessa (BD Biosciences, San Jose, CA). Data were analyzed with FlowJo software (BD Biosciences).

#### FTY720 treatment

To block migration of lymphocytes from lymphoid organs into the skin, mice were treated with FTY720 (Sigma) same as previously reported.^46^ Briefly, FTY720 was dissolved in dimethyl sulfoxide (DMSO) and further diluted in PBS for administration. Mice received intraperitoneal injection of FTY720 at a dose of 1 mg/kg on the day prior to the first *C. auris* application and then every day until the end of the experiment. Control mice were injected with PBS on the same schedule.

#### Injection of neutralizing anti-IL-17A antibodies

In the one-time *C. auris* topical application scheme, mice were intraperitoneally injected with neutralizing anti-IL-17A antibodies (5 mg/kg each injection) one day before and 3 days after *C. auris* application. Mice were euthanized for analyses on day 6 after *C. auris* application.

### Quantification and statistical analysis

Graphs were created and analyzed using Graphpad Prism software 8.0. Statistical differences were determined using student *t* test. The data are presented as mean ± SD.
