# Supplementary material for: Skin-resident T cells play an important role in controlling skin colonization of Candidozyma (Candida) auris
Source: iScience. 2026 Apr 24;29(6):115862. doi: 10.1016/j.isci.2026.115862 (PMC13194182; doi:10.1016/j.isci.2026.115862)
Supplement: Document S1. Figures S1 and S2 [file mmc1.pdf]

iScience, Volume *29*

Supplemental information

Skin-resident T cells play an important role in controlling skin colonization of *Candidozyma (Candida) auris*

Jiajia Xie, Liping Yan, David Kadosh, and Na Xiong

SUPPLEMENTARY FIGURES

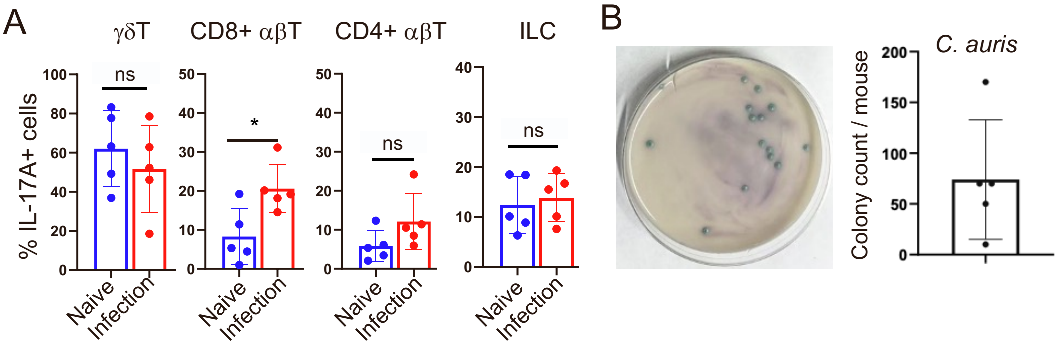

Figure S1. Activation of IL-17A^{+} T cells is associated with controlling topical colonization of *C. auris* in the skin of WT mice. (**A**) Percentages of skin γδT, CD8^{+} αβT, CD4^{+} αβT cells and ILCs that express IL-17A in naïve and 4x repeated *C. auris*-colonized (infected) WT mice. ns: not significantly different (P>0.05), *P<0.05. One dot is of one mouse. Statistical significance was determined by unpaired T test. (**B**) A representative image of colonies of *C. auris* of the skin digests of mice after 4x repeated *C. auris* topical applications in a CHROMagar *Candida* plate colony formation assay. Average numbers of colonies per mouse are shown in the graph on the left. One dot is of one mouse. N=5. Data were compiled of 2 separate experiments.

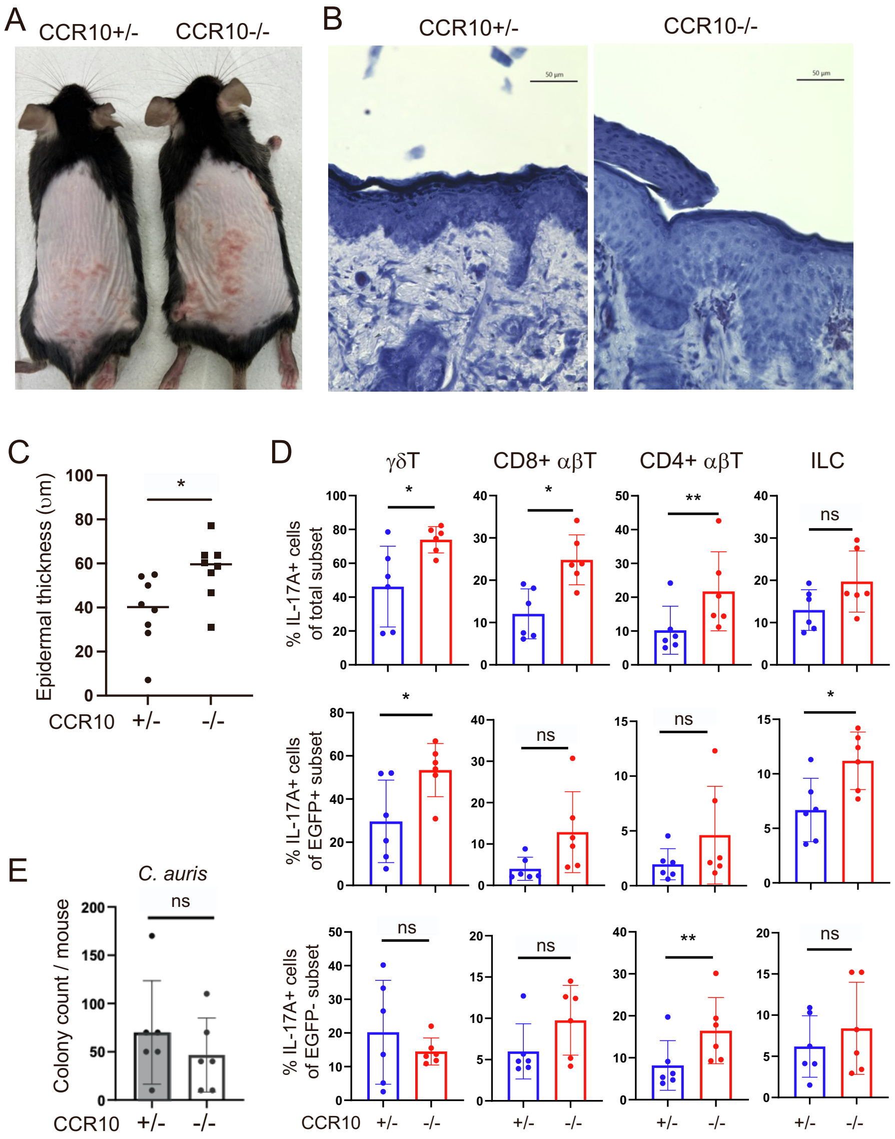

Figure S2. Increased IL-17A^{+} T cells compensate for impaired regulation of *C. auris* colonization by tissue-resident T cells in CCR10-KO mice. (**A**) Images of skin of CCR10$^{+/EGFP}$ and CCR10$^{EGFP/EGFP}$ mice 6 days after 1x *C. auris* application. **(B)** Microscopic images of H&E-stained skin sections of CCR10$^{+/EGFP}$ and CCR10$^{EGFP/EGFP}$ mice 6 days after 1x *C. auris* application. **(C)** Comparison of the thickness of skin epidermis of CCR10$^{+/EGFP}$ and CCR10$^{EGFP/EGFP}$ mice 6 days after 1x *C. auris* application. One dot is of one mouse. N=8. Data were compiled of three separate experiments. **(D)** Percentages of total (top row), EGFP(CCR10)$^{+}$ (middle), and EGFP(CCR10)$^{-}$ (bottom) skin γδT, CD8^{+} αβT, CD4^{+} αβT cells and ILCs that express IL-17A in CCR10$^{+/EGFP}$ and CCR10$^{EGFP/EGFP}$ mice 13 days after the first application of the 4x *C. auris* application scheme. **(E)** Average numbers of colonies of *C. auris* in the skin of CCR10$^{+/EGFP}$ and CCR10$^{EGFP/EGFP}$ mice 13 days after the first application of the 4x *C. auris* application scheme. ns: not significantly different (P>0.05), *P<0.05, ** P<0.01. One dot is of one mouse. N=6. Data were compiled of three separate experiments. Statistical significance was determined by unpaired T test.
